# The Predictive Value of Carotid Ultrasonography With Cardiovascular Risk Factors—A “SPIDER” Promoting Atherosclerosis

**DOI:** 10.3389/fcvm.2021.706490

**Published:** 2021-08-10

**Authors:** Hongwei Li, Xiaolin Xu, Baoming Luo, Yuling Zhang

**Affiliations:** ^1^Department of Cardiology, Sun Yat-sen Memorial Hospital, Sun Yat-sen University, Guangzhou, China; ^2^Guangdong Province Key Laboratory of Arrhythmia and Electrophysiology, Guangzhou, China; ^3^Department of Ultrasound, Sun Yat-sen Memorial Hospital, Sun Yat-sen University, Guangzhou, China

**Keywords:** carotid ultrasonography, carotid intima-media thickness, carotid plaque, cardiovascular risk prediction, cardiovascular risk factors, ultrasound, atherosclerosis, vascular disease, cardiovascular disease, peripheral vascular disease.

## Abstract

Insufficient recommendations do not support the clinical use of carotid ultrasonography for further risk stratification in moderate-to-high risk patients with cardiovascular disease (CVD). A literature review was performed to assess six aspects of the research progress and limitations of carotid ultrasonography and carotid atherosclerosis-related risk factors: (1) structures of the carotid intima and media; (2) plaques; (3) inflammation; (4) dynamics of carotid blood flow; (5) early detection and intervention; and (6) risk factors for CVD. Although carotid intima-media thickness and carotid plaques are well-acknowledged independent predictors of CVD risk, normative and cut-off values are difficult to define due to the heterogeneous measurements reported in previous studies. Plaque properties, including location, number, density, and size, become more important risk predictors for cardiovascular disease, but a better approach for clinical use needs to be further established. Three-dimensional ultrasound and contrast-enhanced ultrasound are promising for promoting risk stratification with more details on plaque morphology. Moreover, inflammatory diseases and biomarkers should be evaluated for a full assessment of the inflammatory burden for atherosclerosis. Carotid flow velocity is not only an indicator for stenosis but also a potential risk predictor. Carotid atherosclerosis should be detected and treated early, and additional clinical trials are needed to determine the efficacy of these measures in reducing CVD risk. Cardiovascular risk factors tend to affect carotid plaques, and early treat-to-target therapy might yield clinical benefits. Based on the aforementioned six aspects, we consider that these six important factors act like a “SPIDER” spinning the web of atherosclerosis; a timely comprehensive assessment and intervention may halt the progression to CVD. Carotid ultrasound results should be combined with other atherosclerotic factors, and a comprehensive risk assessment may help to guide cardiovascular prevention decisions.

## Background

Carotid ultrasonography is a non-invasive, non-radioactive and reproducible imaging method used to detect carotid atherosclerosis and screen high-risk patients for cardiovascular disease (CVD). The carotid intima-media thickness (CIMT) is recommended to be measured in asymptomatic patients at intermediate risk for further risk stratification (1). However, the inconsistency in cut-off values and additive values used in cardiovascular risk prediction models limit the clinical application of CIMT (2, 3). Although carotid plaques are independently associated with an increased CVD risk and are recommended to be screened in patients with diabetes for a cardiovascular risk evaluation, the current method used to detect the presence of carotid plaques does not comprehensively consider their morphological properties (4–6). Furthermore, evidence that early carotid atherosclerosis interventions are beneficial is lacking (1). Despite the large body of research, no individual parameters of carotid ultrasonography are sufficient for determining an accurate prediction of the cardiovascular risk in asymptomatic patients. Insufficient recommendations limit the clinical use of carotid ultrasonography for cardiovascular risk evaluations as a primary preventative measure. A recent review focusing on the usefulness of carotid ultrasonography for risk stratification of cerebral and cardiovascular disease suggests the need to consider various aspects of carotid ultrasound imaging (7). In addition to carotid ultrasonography itself, there are other atherosclerotic factors affecting carotid atherosclerosis and cardiovascular events. Hence, we further propose a combination of carotid ultrasound, clinical condition, and laboratory tests to comprehensively evaluate the future risk of CVD.

In this review, we summarize the research progress, predictive value and limitations of carotid ultrasonography in determining the structure of the carotid intima and media, plaques and carotid flow velocity, and discuss other carotid atherosclerotic factors, including inflammation, early detection and intervention, and traditional CVD risk factors. A comprehensive cardiovascular assessment based on carotid ultrasonography with other atherosclerotic factors is important for risk stratification and medical decisions.

## Direct Parameters of Carotid Ultrasonography

### Structures of the Carotid Intima and Media

#### CIMT

An abnormal increase in CIMT reflects the progression of carotid atherosclerosis, which is detected clearly by ultrasound. The CIMT has been suggested to be an independent predictor of the risk of incident CVD in most studies (8–10). Moreover, a slower CIMT progression caused by therapeutic intervention could predict the degree of CVD risk prediction (11). It is measured between the lumen-intima and the media-adventitia interfaces at the far wall of carotid arteries on ultrasound images, which show an obvious “double-line” sign. Changes in the intima and media have been further specified, as the intima layer is thicker and the media layer is thinner in patients with CVD than in healthy subjects (12). There were relatively few small-scale cross-sectional studies calculating the age-specific normal value of CIMT in healthy individuals, but the measurement methods and normative value of CIMT were not identical (13, 14). To date, a stable normative value for CVD risk prediction has not yet been defined (2).

The CIMT measurements, endpoint events and cut-off values for the CIMT used in large population-based prospective studies are summarized in Table 1. The discrepancies between studies concern five aspects: (1) the sites of CIMT measurement; (2) the CIMT parameters used for statistical analysis; (3) the endpoints of each study; (4) whether carotid plaques are included; and (5) the cut-off values used for predicting CVD risk.

**Table 1:** Prospective studies with a large general population that assessed the association between carotid intima-media thickness (CIMT) and cardiovascular risk.

**Study**	**Year**	**Sample size**	**Age (years)**	**Follow-up**	**Measurements**	**Primary endpoints**	Hazard ratio* (95% CI)
KIHD (5)	1991	1,288	42–60	1 month−2.5 years	Mean of the maximal CCA-IMT, far wall, bilateral, three repeated measures, mineralized plaque not included	AMI	2.17 (1.08–4.26) per 0.1 mm
ARIC (8)	1997	12,841	45–64	Median 5.2 years	Mean CIMT of CCA + BIF + ICA, far wall, bilateral	MI, CHD death	IMT ≥ 1 mm: women: 5.07 (3.08–8.36), men: 1.85 (1.28–2.69); The third tertile vs. the first tertile: women: 6.69 (3.01–14.89), men: 2.88 (1.91–4.34)
CHS (9)	1999	4,476	72.5 ± 5.5	Median 6.2 years	Mean of the maximal CCA/ICA-IMT and the average CIMT, near and far walls, bilateral, three repeated measures for ICA, focal plaque included	MI, stroke	Relative risk per 1 SD increase: CCA-IMT: 1.27 (1.17–1.38); ICA-IMT: 1.30 (1.20–1.41); average IMT: 1.36 (1.25–1.47); The top quintile vs. the bottom quintile: CCA-IMT: 2.22 (1.58–3.13); ICA-IMT: 2.47 (1.59–3.85); average IMT: 3.15 (2.19–4.52)
Rotterdam study (15)	2004	6,389	69.3 ± 9.2	7–10 years	Mean of the maximal CCA-IMT, near and far walls, bilateral	MI	IMT ≥ 1.12 mm: 1.95 (1.19–3.19)
MDCS (16)	2005	5,163	45–64	Median 7 years	CCA-IMT, far wall, right	MI, IHD	IMT per 1SD increase: 1.23 (1.07–1.41); The top quintile vs. the bottom quintile: 2.76 (1.05–7.25)
CAPS (17)	2006	5,056	50.1 ± 13.1	Mean 4.2 years	Mean CCA/BIF/ICA-IMT, far wall, bilateral	MI, stroke, death	IMT per 1 SD increase: CCA: 1.17 (1.08–1.26); BIF: 1.14 (1.05–1.24); ICA: 1.09 (1.01–1.18); The top quartile vs. the bottom quartile: CCA: 1.85 (1.09–1.35); BIF: 1.27 (0.80–1.99); ICA: 1.25 (0.84–1.86)
EAS (18)	2007	1,007	69.4 ± 5.6	12 years	Maximal CCA -IMT, far wall, bilateral	MI, angina, stroke, IC	Odd ratios for MI/stoke with IMT ≥ 0.9 mm: 1.59 (1.07–2.37)
Tromsø study (19)	2007	6,226	55–74	Mean 5.4 years	Mean CCA-IMT and mean IMT, near and far walls for the CCA and far walls for the carotid bulb, right, plaque included	MI	Relative risk: the top quartile vs. the bottom quartile: 1.73 (0.98–3.06) for men and 2.86 (1.07–7.65) for women
CCCC (20)	2008	2,190	≥35	Median 10.5 years	Mean of the maximal CCA-IMT, far wall, bilateral	MI, CHD death, revascularization	Relative risk for CHD: IMT per 1 SD increase: 1.38 (1.12–1.70)
Cournot et al. (21)	2009	2,561	51.6 ± 10.5	Median 6 years	Mean CCA-IMT, far wall, bilateral, 3 times, plaque not included	AMI, angina, cardiac death, sudden death	IMT > 0.63: 2.26 (1.35–3.79)
Three-city study (22)	2011	5,895	73.3 ± 4.9	Median 5.4 years	Mean CCA-IMT, far wall, bilateral, plaque not included	MI, angina, CHD death, revascularization	IMT per 1SD increase: 0.8–1.1; The top quintile vs. the bottom quintile: 0.8 (0.5–1.2)
Framingham offspring study (23)	2011	2,965	58 ± 10	Average 7.2 years	Mean CCA-IMT and maximal ICA-IMT, far wall, bilateral, end-diastole, plaque not included	MI, angina, HF, CHD death, stroke, IC	IMT per 1 SD increase: CCA: 1.13 (1.02–1.24); ICA: 1.21 (1.13–1.29); IMT per 1 mm increase: CCA: 2.46 (1.18–5.13) ICA: 1.26 (1.16–1.36)
FATE (24)	2011	1,574	49.4 ± 9.9	Mean 7.2 years	Mean of the maximum CCA-IMT, far wall, right, at least 3 repeated measures	MI, RSCA, revascularization, SVD	IMT per 1 SD increase: 1.45 (1.15–1.83)
IMPROVE (25)	2012	3,703	Mean 64.2	Median 36.2 months	Mean and maximal CCA/BIF/ICA-IMT, far wall, bilateral, 3 angles, plaque included	MI, angina, HF, SCD, stroke, IC, revascularization	Mean IMT per 1SD increase: CCA: 1.31 (1.14–1.49); BIF: 1.24 (1.08–1.44); ICA: 1.27 (1.11–1.44); Maximal IMT per 1SD increase: CCA: 1.27 (1.12–1.44); BIF 1.26 (1.08–1.46); ICA: 1.30 (1.14–1.50)
MESA (26)	2012	1,330	63.8 ± 9.5	Median 7.6 years	Mean of the maximal CCA-IMT, far wall, right, plaque excluded	MI, angina, CHD death, RSCA, revascularization	IMT per 1 SD increase: 1.17 (0.95–1.45)
Suzuki et al. (ARIC + CHS) (27)	2020	20,862	ARIC: 54.2 ± 5.8 CHS: 72.8 ± 5.5	23.5 years in ARIC 13.1 years in CHS	ARIC: Mean and maximum CIMT of CCA + BIF + ICA, far wall, bilateral CHS: Mean of the maximal CCA/ICA-IMT and the average CIMT, near and far walls, bilateral	SCD	The fourth quartile vs. the first quartile of maximal CIMT: 1.75 (1.22–2.51)
CIRCS (28)	2020	2,943	40–75	Median 15.1 years	Mean of the maximal CCA/ICA-IMT and the average CIMT, near and far walls, bilateral	CHD and stroke	The highest quartile vs. the lowest quartile of maximal CCA-IMT: 2.11 (1.44–3.11) The highest quartile vs. the lowest quartile of maximal ICA-IMT: 1.78 (1.20–2.62)

*The measurements of CIMT are reported in the following order: the parameters, near/far wall, left/right side (if specified), number of repeated measures, systolic/diastolic phase and whether plaque was included*.

* *Hazard ratios were adjusted by age, sex, other traditional risk factors and medications provided by each study*.

*AMI, acute myocardial infarction; ARIC, the Atherosclerosis Risk in Communities Study; BIF, carotid bifurcation; CAPS, the Carotid Atherosclerosis Progression Study; CCA, common carotid artery; CCCC, the Chin-Shan Community Cardiovascular Cohort Study; CHD, coronary heart disease; CHS, the Cardiovascular Health Study; CI, confidence interval; EAS, the Edinburgh Artery Study; FATE, the Firefighters and Their Endothelium Study; HF, heart failure; KIHD, the Kuopio Ischemic Heart Disease Risk Factor Study; IC, intermittent claudication; ICA, internal carotid artery; IHD, ischemic heart disease; IMPROVE, the IMT-Progression as Predictors of Vascular Events in a High-Risk European Population Study; MESA, the Multi-Ethnic Study of Atherosclerosis; MDCS, the Malmo Diet and Cancer Study; MI, myocardial infarction; RSCA, resuscitated cardiac arrest; SCD, sudden cardiac death; SVD, systematic vascular disease; mm, millimeter; SD, standard deviation*.

Most studies measured the CIMT at the far wall of bilateral common carotid arteries (CCAs), but some studies measured the CIMT at both the near and far walls, at the carotid bifurcations (BIFs) and internal carotid arteries (ICAs), or only at the right CCA; measurements were recorded at three different angles or were repeated three times. The maximal CCA-IMT (18); the mean maximal CIMT (5, 9, 15, 20, 24, 26); the mean CIMT normalized by the IMT of the bilateral CCAs, BIFs, and ICAs (8); and the mean CCA-IMT and ICA-IMT of the bilateral carotid arteries (10, 17, 22, 25, 26) have been separately used for establishing distinct risk prediction models. For example, Chambless et al. (8) defined the mean CIMT as the average of the bilateral CCA-IMT, BIF-IMT, and ICA-IMT, while Baldassarre et al. (25) and O'Leary et al. (9) reported different values for the mean CIMT of the bilateral CCAs and ICAs.

Growing evidence suggests that CIMT can predict CVD risk partly because of the inclusion of plaques (22), which might magnify the measurement of the IMT and elicit false positive associations with CVD risk. Studies have reported the mean CCA-IMT, excluding plaques, but did not identify an association between the CCA-IMT and risk of incident CVD (22, 26, 29). Another source of heterogeneity was the arbitrary cut-off value used to predict the risk of the cardiovascular endpoints. For instance, the top quintile of the mean CCA-IMT was 1.18 mm in the Cardiovascular Health Study (9), while the top quintile of the mean CCA-IMT was 0.805 mm in the three-city study (22). Such heterogeneity might affect the comparison and synthesis of CIMT results.

**Figure 1:**
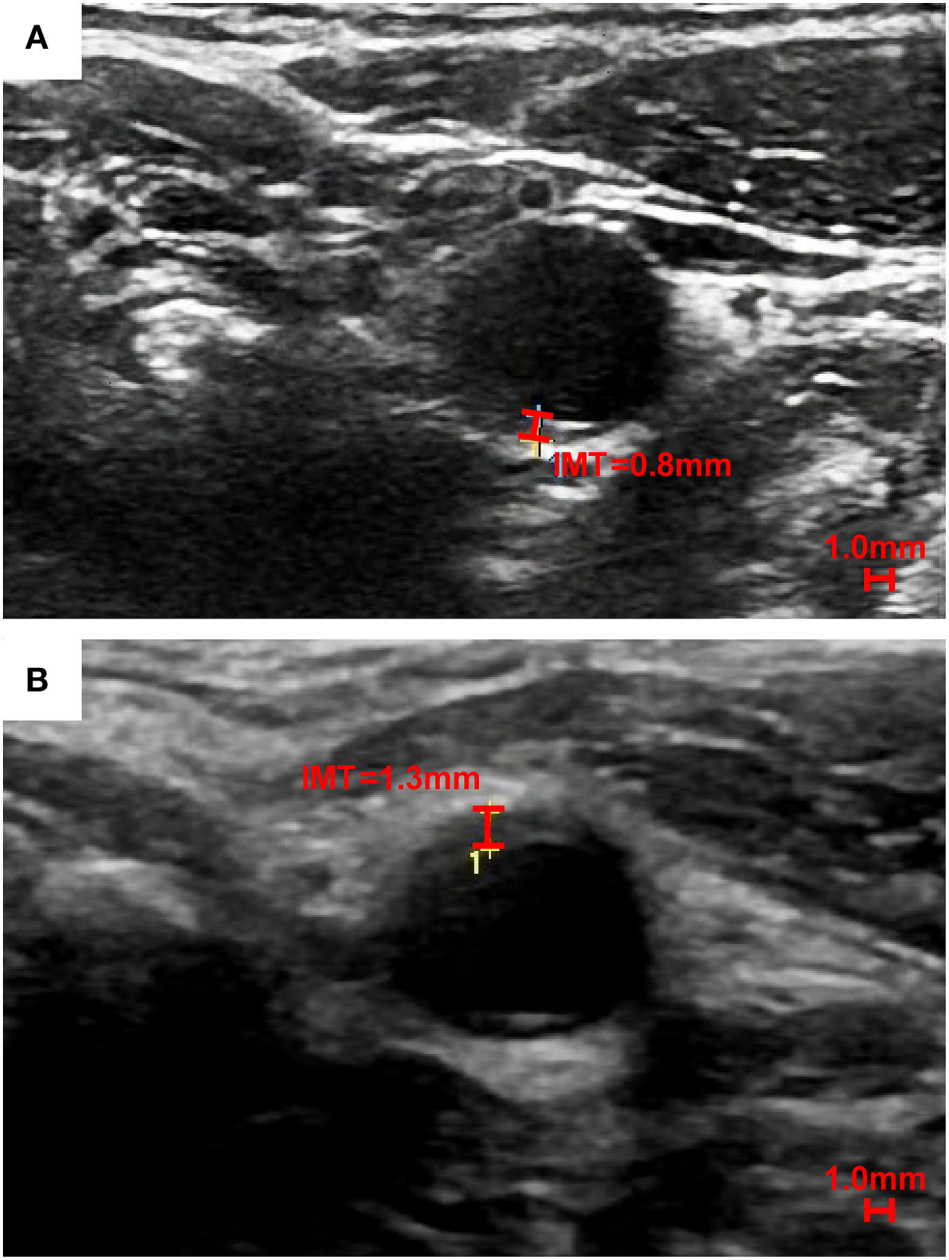
A single-angle cross-sectional scan of the near wall and far wall CIMT of the left CCA. To better show the distinction between the near wall and far wall of common carotid artery, we choose the cross section. The far wall CIMT of a 50-year-old Chinese man is 0.8 mm in **(A)**, while the near wall CIMT of a 41-year-old Chinese man is thicker (1.3 mm) than the far wall CIMT in **(B)**.

The consensus for the use of carotid ultrasound to evaluate CVD risk and identify subclinical vascular disease issued by the American Society of Echocardiography (ASE) in 2008 established a standard method for CIMT assessment. It is categorized into four parts: (1) a cross-sectional scan for an overview of the arterial wall structure; (2) a Doppler ultrasound scan for identifying significant stenosis; (3) three-angle scans (anterior, lateral, and posterior) for the identification and description of plaques at the near and far walls of bilateral CCAs, bulbs, and ICAs; and (4) three-angle images of the “double line” sign for CIMT measurements, with the distal 1 cm of each CCA gated by the optimized R-wave (4). The accuracy of far-wall CCA-IMT measurements has been validated by the absence of a significant difference from *in vitro* specimens (30), while the near-wall measurements are less accurate due to liable artifacts. Three-angle scans of the CCAs help prevent an irregular CIMT from being inadequately evaluated without considering the near-wall CIMT. For instance, the far-wall CIMT of a 50-year-old man is clearly measured to be 0.8 millimeters (mm) in Figure 1A, while the CIMT of another 41-year-old man was significantly thicker, measuring 1.3 mm at the near wall in Figure 1B. In addition to CCA-IMT, significantly thicker CIMT values at carotid bulbs and ICAs should not be neglected, especially in patients with normal CCA-IMT values, to avoid underestimations of cardiovascular risk. The Mannheim CIMT and Plaque Consensus states that CIMT can be assessed at carotid bulbs and ICAs (31). A CIMT ≥75th percentile after adjusting for the patient's age, ethnicity, and sex is recommended as an indication of an increased CVD risk.

A comprehensive meta-analysis of 16 prospective studies performed by the PROG-IMT collaboration revealed a positive association between the mean CCA-CIMT and a 16% increase in cardiovascular risk but no association between CIMT progression and cardiovascular events (32); moreover, the identification of meaningful normative values is difficult due to the substantial heterogeneity in the percentiles of CIMT reported across studies (2). In addition, another meta-analysis challenged the additive value of CIMT, as it showed only a 0.8% net reclassification improvement (NRI) after it was added to the 10-year Framingham risk score (FRS)-based risk prediction model (3). The challenge to the predictive value of CIMT progression and the additive value of CIMT to the FRS reduce the priority to measure the CIMT in cardiovascular risk assessments (33, 34). Interestingly, recent research using two cohorts with 20,862 participants from the ARIC study and the CHS study revealed a significantly positive association between CIMT and sudden cardiac death (SCD) during at least 13.1 years of follow-up (27). The long-term predictive value of CIMT for SCD may be better than the unsatisfactory predictive ability of 10-year total cardiovascular risk.

In short, far-wall CIMT is a useful independent predictor for CVD risk, with good reproducibility and accuracy. Due to the heterogeneity of CIMT measurements, CIMT results must be combined with other atherosclerotic markers instead of using carotid ultrasound alone (18, 35). Furthermore, the lifetime predictive value of the CIMT in young adults is worth exploring, regardless of whether the 10-year risk predictive value is low.

#### Carotid Artery Diameters

The CCA diameter, which is affected by the CIMT and carotid plaques, increases in individuals with carotid atherosclerosis and is measured transversely at a plaque-free site (36). An increased carotid lumen diameter was demonstrated to be associated with increased all-cause mortality (37), but its correlation with incident cardiovascular disease was uncertain. The interadventitia CCA diameter (ICCAD) is easier to detect than the lumen diameter, and an increase in the ICCAD exhibits additive predictive value for composite cardiovascular events (25). A meta-analysis of four cohort studies also reported that patients with a CCA diameter >8 mm had a higher risk of total CVD than patients with a diameter <7 mm (38). However, direct evidence that the ICCAD or the CCA diameter predicts the risk of coronary heart disease is unavailable (38). Carotid artery diameters represent the structural and functional changes induced by atherosclerosis and may be associated with an increased cardiovascular risk. Carotid arterial diameters should not be neglected in risk assessments.

### Plaques

#### The Presence of Carotid Plaques

In people aged 30–79 years, the global prevalence of an increased CIMT is estimated to be 27.6% and the global prevalence of the presence of carotid plaques is estimated to be 21.1% in 2020. The percent increase in both an increased CIMT and the presence of carotid plaques is >50% in 2020 (39). The carotid atherosclerotic burden is significantly increased worldwide. Accumulating evidence from prospective studies has identified the presence of carotid plaques as a strong independent CVD risk factor, with significant additive value for risk prediction models (10, 40). Carotid plaques are defined as having a focal thickness that is at least 50% greater than that of the surrounding wall or a focal thickness distinct from the adjacent boundary greater than 1.5 mm, with protrusion into the lumen, which easily occurs at carotid bulbs. Carotid plaques are classified into three grades according to the up-to-date recommendations for the assessment of carotid arterial plaque by ultrasound from ASE: grade I is defined as protuberant plaques with CIMT <1.5 mm, grade II as either protuberant or diffuse plaques with CIMT ≥1.5 mm but <2.5 mm, and grade III as either protuberant or diffuse plaques with CIMT ≥2.5 mm (41). Plaques are recommended to be detected at both the near and far walls of bilateral CCA, carotid bulb and ICA segments, and the presence of carotid plaques after adjustment for the patient's age, sex, and ethnicity implicates an increased CVD risk (4, 42). Carotid atherosclerosis including carotid plaques is a strong cardiovascular predictor, even among patients with previous myocardial infarction or previous stroke (43).

A summary of the large prospective studies on the association between carotid plaques and CVD risk is shown in Table 2. Heterogeneity in plaque measurements mainly exists due to heterogeneity in three aspects: (1) the site at which plaques are detected; (2) the parameters of interest for plaques; and (3) the definition of plaques. Most of the studies detected plaques at bilateral CCAs, carotid bulbs, and ICAs (15). A few studies detected carotid plaques in the bilateral CCAs and bulbs (5), bilateral CCAs and ICAs (19, 21), and only the right carotid arteries (16).

**Table 2:** Prospective studies with a large general population that assessed the association between carotid plaques and cardiovascular risk.

**Study**	**Year**	**Sample size**	**Age (years)**	**Follow-up**	**Measurements**	**Primary endpoints**	Hazard ratio*
KIHD (5)	1991	1,288	42–60	1 month−2.5 years	Bilateral CCAs + carotid bulbs, the presence of plaques, a focal area with mineralization or protrusion into the lumen	AMI	Small plaque: 4.15 (1.15–11.47); Stenotic plaque^†^: 6.71 (1.33–33.91)
Rotterdam study (15)	2004	6,389	69.3 ± 9.2	At least 7 years	Bilateral CCAs + carotid bulbs + ICAs, the presence of plaques and plaque score, a focal area with protrusion into the lumen	MI	Plaque score ≥ 3 points: 1.38 (1.27–2.62)
MDCS (16)	2005	5,163	45–64	Median 7 years	Right CCA + carotid bulb + ICA + ECA, the presence of plaques and plaque score, a focal thickening of IMT > 1.2 mm	MI, IHD	Plaque score per 1 SD increase: 1.39 (1.10–1.78)
CHS (44)	2007	5,020	72.6 ± 5.5	Median 11 years	Bilateral CCAs + ICAs, classification of plaques, the largest focal lesion classified by surface characteristics, echogenicity, and texture	MI, stroke, cardiovascular death, all-cause death	High-risk plaque^‡^: 1.38 (1.14–1.67)
Tromsø study (19)	2007	6,226	55–74	Mean 5.4 years	Bilateral CCAs + carotid bulbs + ICAs, the presence of plaques + plaque echogenicity (grade 1–4) + plaque area, a focal area with protrusion into the lumen	MI	Relative risk according to plaque area: the top tertile vs. no plaque: 1.56 (1.04–2.36) for men; 3.95 (2.16–7.19) for women; Echogenicity: the bottom tertile vs. no plaque: 1.08 (0.68–1.70) for men; 2.79 (1.45–5.37) for women
NOMAS (45)	2007	1,118	68 ± 8	Mean 2.7 years	Bilateral CCAs + ICAs, the presence of plaques and calcified plaque, a focal area with protrusion 50% greater than the surrounding wall and plaques with acoustic shadowing were calcified	MI, stroke, vascular death	Calcified plaques vs. no plaque: 2.4 (1.0–5.8)
NOMAS (6)	2008	2,189	68 ± 10	Mean 6.9 years	Bilateral CCAs + BIFs + ICAs, MCPT, a focal area with protrusion 50% greater than the surrounding wall	MI, stroke, vascular death	MCPT ≥ 1.9 mm vs. no plaque: 1.48 (1.05–2.10);
CCCC (20)	2008	2,190	≥35	Median 10.5 years	Bilateral CCAs + carotid bulbs + ICAs + ECAs, the severity of plaque score	MI, CHD death, revascularization	Relative risk for CHD: 1.15 (1.07–1.24) per 1 increase in plaque score
Cournot et al. (21)	2009	2,561	51.6 ± 10.5	Median 6 years	Bilateral CCAs + ICAs, the presence of plaques, a focal area with hyperechogenicity or protrusion into the lumen	AMI, angina, cardiac death, sudden death	2.81 (1.84–4.29)
Three-city study (22)	2011	5,895	73.3 ± 4.9	Median 5.4 years	Bilateral CCAs + BIFs + ICAs, the presence of plaques, a focal area with protrusion into the lumen for at least 50% greater than the surrounding vessel wall	MI, angina, CHD death, PCI, CABG	Plaques at ≥ 2 sites vs. no plaque: 2.2 (1.6–3.1)
Framingham offspring study (23)	2011	2,965	58 ± 10	Average 7.2 years	Bilateral ICA, the presence of plaques, an area of IMT ≥ 1.5 mm	MI, angina, CHD death, stroke, IC, HF	1.92 (1.49–2.47)
MESA (10)	2013	6,562	61.1 ± 10.2	Mean 7.8 years	Bilateral CCAs + BIFs + ICAs, the presence of plaques, a focal area with protrusion into the lumen	MI, angina, CHD death, RSCA	1.45 (1.20–1.76)
High-risk plaqueBioImage (29)	2017	5,808	Average 69	Median 2.7 years	Bilateral carotid arteries, MCPT and total plaque area (mm^2^), a focal protrusion ≥ 0.5 mm or 50% of the surrounding wall; or IMT > 1.5 mm	MI, stroke, cardiovascular death	The top tertile vs. the bottom tertile: MCPT: 1.96 (0.91–4.25); Total plaque area: 2.36 (1.13–4.92)
MESA (46)	2017	4,955	61.6 ± 10.1	Median 11.3 years	Bilateral CCAs + BIFs + ICAs, plaque score, a focal thickness of IMT > 1.5 mm or > 50% of the surrounding wall	MI, angina, CHD death, RSCA, stroke	Plaque score per 1 SD increase: 1.27 (1.16–1.40)
MESA (47)	2018	2,706	65.4 ± 9.6	Mean 13.3 years	Bilateral CCAs + BIFs + ICAs, plaque score and total plaque score and greyscale plaque features	MI, angina, CHD death, RSCA, stroke	Total plaque area per 1 SD increase: 1.23 (1.11, 1.36); carotid plaque score per 1 SD increase: 1.33 (1.18–1.49)
Suzuki et al. (ARIC + CHS) (27)	2020	20,862	ARIC: 54.2 ± 5.8 CHS: 72.8 ± 5.5	23.5 years in ARIC 13.1 years in CHS	ARIC: Bilateral CCAs + BIFs + ICAs, the presence of plaques, two of the following criteria: CIMT > 1.5 mm/a focal protrusion into the lumen/brighter echoes than adjacent boundaries CHS: Bilateral CCAs + ICAs, the presence of high risk and intermediate risk plaques^‡^^#^	SCD	ARIC: 1.37 (1.13–1.67) CHS: 1.32 (1.04–1.68)
CIRCS (28)	2020	2,943	40–75	Median 15.1 years	Bilateral ICAs, the presence of homogeneous or heterogeneous plaques, a focal thickness of IMT ≥ 1.5 mm	CHD and stroke	1.71 (1.25–2.35)

*The measurements of the carotid artery are reported in the following order: the segments, parameters, and definitions of plaques*.

* *Hazard ratios were adjusted by age, sex, other traditional risk factors and medications provided by each study*.

† *A plaque with ≥20% diameter stenosis was classified as stenotic plaque*.

‡ *High-risk plaque was defined as plaques with markedly irregular or ulcerated surfaces or hypodense or heterogeneous plaques that occupied 50% of the total plaque volume*.

# *Intermediate- risk plaque was defined as hyperdense, calcified, or homogeneous plaque or those with a mildly irregular surface*.

*AMI, acute myocardial infarction; BIF, carotid bifurcation; CCA, common carotid artery; CCCC, the Chin-Shan Community Cardiovascular Cohort Study; CHD, coronary heart disease; CHS, the Cardiovascular Health Study; CI, confidence interval; CIRCS, the Circulatory Risk in Communities Study; ECA, external carotid artery; KIHD, the Kuopio Ischemic Heart Disease Risk Factor Study; HF, heart failure; IC, intermittent claudication; ICA, internal carotid artery; IHD, ischemic heart disease; mm, millimeter; MESA, the Multi-Ethnic Study of Atherosclerosis; MCPT, the maximum carotid plaque thickness; MDCS, the Malmo Diet and Cancer Study; MI, myocardial infarction; NOMAS, the Northern Manhattan Study; RSCA, resuscitated cardiac arrest; SD, standard deviation*.

The parameters of interest for carotid plaques also varied across studies. Despite the presence of plaques, most studies chose plaque scores to predict the risk (16, 18, 20, 36), while some studies collected data on plaque echogenicity, plaque texture, plaque surface, and plaque area (9, 19, 29). In addition, early studies defined plaques as a focal area with protrusion into the lumen without a cut-off standard for CIMT, which might lead to overestimations of the predictive value of CIMT. The heterogeneity of plaque parameters also increased the difficulty of identifying a stable cut-off value for plaque number or properties to predict the cardiovascular risk. Uniform quantification of carotid plaques may help to better establish the cut-off value for parameters of carotid plaques in cardiovascular risk prediction.

#### Plaque Scores

Various scoring systems with satisfactory predictive ability have been developed to quantitatively measure carotid plaques (Table 3). In the Rotterdam study, the plaque score was computed as the total number of sites where plaques occurred in bilateral CCAs, BIFs, and ICAs, with a total score of 6 points (15). A similar score was calculated for the near and far walls of the bilateral CCAs, BIFs, and ICAs, with a total score of 12 points, in the MESA study (46). Plaque scores reflecting the severity of plaques in carotid arteries also showed a significant association with cardiovascular events (16, 20). Handa et al. (48) reported an algorithm that calculated the sum of the bilateral maximal thickness of each plaque in four segments, which was associated with the severity of coronary artery lesions (50). Prati et al. (49) established a scoring system consisting of four parts: the degree of stenosis, echogenicity, heterogeneity of the texture, and surface characteristics of each plaque. These quantitative plaque scoring systems are considered to improve the evaluation of plaque severity and future cardiovascular risk.

**Table 3:** Differences between the carotid plaque scoring systems.

**Study**	**Scoring system**
Rotterdam study (15) and MESA (46)	The sum of the sites with plaque in (the near and far walls of) the CCA, BIF, and ICA
MDCS (16)	A scale dependent on the severity of BIF: 0 = normal; 1 = one small plaque (<10 mm^2^); 2 = small plaques ≥2; 3 = one large plaque (>10 mm^2^); 4 = one large plaque plus small plaques; 5 = large plaques ≥2 or one plaque with stenosis >50% or circumferent
CCCC (20)	The total grade of each CCA, carotid bulb, ICA and ECA bilaterally: grade 0 = normal; 1= one small plaque (stenosis: <30%); 2 = one medium plaque (stenosis: 30–49%) or multiple small plaques; 3 = one large plaque (stenosis: 50–99%) or multiple plaques with medium plaque ≥1; 4 = occlusion
Handa et al. (48)	The sum of the bilateral maximal thickness of each plaque in the area from the ICA <15 mm proximal to the BIF region to the CCA >30 mm proximal to the BIF region.
Prati et al. (49)	The total score of four parameters: (1) stenosis ≥40% = 1; (2) echogenicity from low (1) to high (3); (3) heterogeneous texture with complex echo pattern = 1; and (4) irregular surface = 1. In addition, the plaque with the highest score was analyzed.

*BIF, carotid bifurcation; CCA, common carotid artery, CCCC, the Chin-Shan Community Cardiovascular Cohort Study; ECA, external carotid artery; ICA, internal carotid artery; MDCS, the Malmo Diet and Cancer Study; MESA, the Multi-Ethnic Study of Atherosclerosis; mm, millimeter*.

#### Plaque Properties

**Figure 2:**
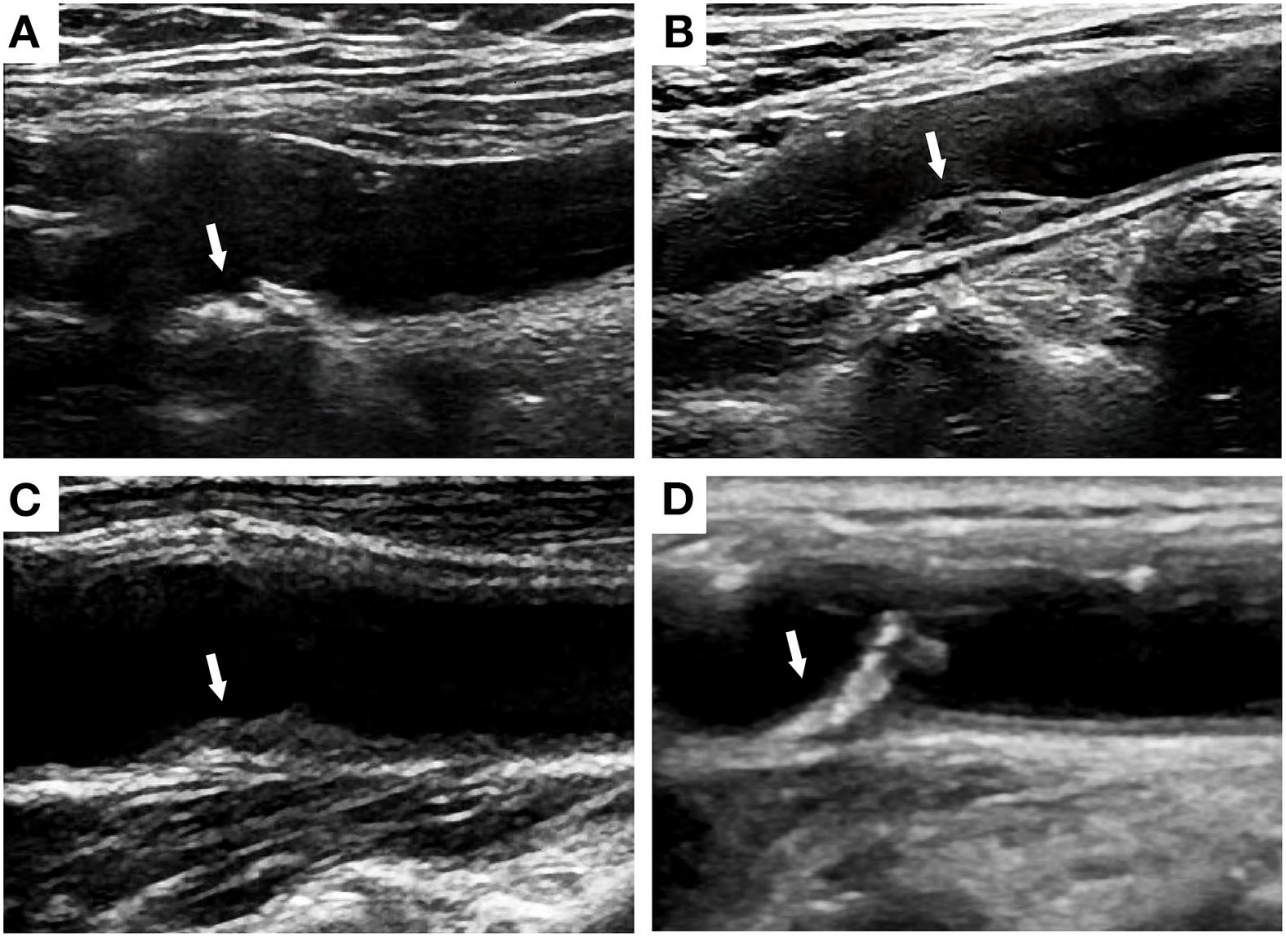
Ultrasound images of hyper-echo **(A)**, low-echo **(B)**, mixed-echo **(C)**, and ulcer plaques **(D)** at the carotid bulbs.

Plaque properties, including the thickness, area, echogenicity and surface characteristics, have attracted the attention of an increasing number of researchers. Rundek et al. measured the maximal carotid plaque thickness, and values greater than the 75th percentile were associated with a 1.48-fold higher risk of combined vascular events (6). In addition, the plaque area also predicts the risks of myocardial infarction (MI), stroke, and cardiovascular death, which are significantly higher in females than in males (19, 29, 47). Carotid plaques are categorized as hyper-echo, low-echo, mixed-echo, and ulcer plaques based on the level of echogenicity (Figure 2). The echogenicity of plaques is positively associated with the density and stability of carotid plaques. Ulcer plaques are more unstable and have a rough surface. Cao et al. (44) defined high-risk plaques as plaques with markedly irregular or ulcerated surfaces or hypodense or heterogeneous plaques that occupy 50% of the total plaque volume, which were related to a 1.38-fold greater risk of composite CVD events. Patients with calcified carotid plaques exhibiting high echogenicity also had a notably higher risk of combined cardiovascular events than patients without plaques (45). In the Circulatory Risk Communities Study, heterogeneous plaques correlated positively with increased risk of total stroke, ischemic stroke, lacunar infarction, coronary artery disease, and total cardiovascular disease. In addition, patients with markedly irregular or ulcerated plaques had a significantly higher risk for coronary artery disease and total cardiovascular disease but not stroke (28). Recording plaque location, thickness, area, and number is recommended for a more precise description of carotid plaques (31). Plaque properties are associated with CVD risk, but additional scientific evidence must be obtained for validation.

Plaque identification by carotid ultrasound is largely based on two-dimensional ultrasound, which cannot display the original three-dimensional structure of carotid plaques. Three-dimensional ultrasound has a wider dynamic scale for measurement of the progression of the plaque area and CIMT (32), which is too small to be detected by a two-dimensional scan. A more accurate measurement of plaque morphology by either single-region or full-vessel protocols by using three-dimensional ultrasound has been recently recommended for the assessment of carotid arterial plaque by ultrasound from ASE. Three-dimensional ultrasound is capable of measuring a specific lesion in all planes, which can avoid missing height or area when it is out of plane by two-dimensional ultrasound imaging (41). A prospective study demonstrated that a three-dimensional plaque volume <0.09 mL can better identify patients with a low risk of coronary artery disease than a two-dimensional plaque thickness <1.35 mm (51). Additionally, 1-year progression of total plaque volume is reported to independently predict cardiovascular events (52). Contrast-enhanced ultrasound is also a novel technology that allows visualization of carotid intraplaque neovascularization and evaluation of carotid plaque vulnerability, and an increased carotid intraplaque neovascularization score is a strong predictor for significant coronary artery disease, with high sensitivity and specificity (53, 54). Superb microvascular imaging ultrasound without contrast is a novel technology using an algorithm to remove clutter and motion wall artifacts under the condition of low-velocity blood flow, and seems to detect carotid intraplaque neovascularization accurately comparable to contrast-enhanced ultrasound (55). Plaque properties obtained by three-dimensional ultrasound, contrast-enhanced ultrasound, and superb microvascular imaging appear to be powerful tools of cardiovascular risk assessment in clinical use, but further studies are necessary to validate precise and practical parameters.

In summary, carotid plaques are more powerful risk predictors than CIMT and should be reported in combination with CIMT (4). For high-risk patients with diabetes whose stratification of CVD risk may be underestimated by traditional risk assessments, carotid plaques should be measured for risk stratification (34).

### Dynamics of Carotid Blood Flow

#### Carotid Flow Velocity

Carotid flow velocity, specifically peak-systolic velocity (PSV), as measured by gray-scale or Doppler ultrasound, is always used to classify ICA stenosis. For instance, a PSV ≥125 cm/s indicates ICA stenosis rate ≥50%, while a PSV ≥230 cm/s indicates >80% ICA stenosis. When the ICA stenosis rate is >90%, PSV is undetectable (56). However, the predictive ability of carotid flow velocity remains unclear. A large follow-up study measured end diastolic velocity (EDV), which was reported to be associated with ischemic stroke. Patients with a low EDV and high IMT exhibited a 2.10-fold higher risk of ischemic stroke than patients with a high EDV and low IMT. However, the predictive value of a low EDV for coronary events has not been validated (57). Another cohort study of patients with hypertension revealed a higher risk of composite cardiovascular events that was related to a PSV/systolic carotid artery diameter < 85.7 s, which provided additive value for risk prediction models (58). Additional studies should be conducted to confirm the association between carotid flow velocity and the risk of coronary heart disease. Carotid flow velocity should not be merely considered as a stratification standard for diameter stenosis.

#### Shear Stress of Carotid Artery Wall

The disrupted and turbulent flow at the stenosis location may promote carotid plaque formation. An asymmetrical distribution of CIMT is closely correlated with hemodynamic changes across the carotid artery, and the highest CIMT was reported to be located at the posteromedial wall of the bifurcation and internal carotid segments and the anterolateral wall of the common carotid segment (59). The maximum wall shear stress appears at the peak of carotid plaques, while the minimum wall shear stress was reported to be located at the place after passing of the peak, which was lower than non-stenotic areas (60). The reduction in carotid endothelial shear stress with age was also an independent predictor of carotid plaque development (61). Goudot et al. suggested that a combination of maximal wall shear stress at the peak of carotid plaque and shear wave elastography texture predicted vulnerable carotid plaques with good performance (62). However, there is no reference value of shear stress for vascular evaluation in clinical practice. Additionally, perivascular adipose tissue also participates in carotid plaque formation induced by disturbed flow in ApoE^−/−^ mice which may be mediated by focal inflammation attenuation (63). Haberka and Gasior provided a novel index of the perivascular adipose index, carotid extra-media thickness (EMT), and found that EMT was positively associated with cardiometabolic risk factors (64). Detection of perivascular adipose tissue by the combination of inflammatory markers and imaging for cardiovascular risk prediction requires further struggles.

## Early Detection and Intervention

CIMT is recommended to be measured in intermediate-risk adults for additional risk stratification. However, whether the early detection of carotid atherosclerosis is beneficial for improving clinical outcomes remains uncertain. There is no strong evidence that suggests the need for therapies for abnormal CIMT (1). Whether extra measures for the early detection and treatment of carotid atherosclerosis are needed is a key question.

A recent randomized controlled study compared the FRS between patients who were informed of their carotid ultrasound results and patients who did not receive their results, and a significant decrease in the FRS from baseline to the 1-year follow-up was unexpectedly observed in the intervention group. Thus, an awareness of subclinical carotid atherosclerosis is beneficial for reducing cardiovascular risk, which might originate from improved compliance with medication and lifestyle modifications (65). In addition, a treat-to-target approach for CVD risk factors, including lifestyle interventions and medications, contributed to slower CIMT progression and lower morbidity related to cardiovascular events compared to usual care in patients with rheumatoid arthritis who did not present with CVD (66). However, in elderly patients with type 2 diabetes and coronary artery disease, the CIMT of patients without identified carotid plaques was reduced by a 1-year exercise training program (67). These studies stressed the importance of early detection and intervention for subclinical carotid atherosclerosis. Carotid ultrasonography may also help to evaluate cerebrovascular and cardiovascular risk without unnecessary invasive examination in low-risk patients with infectious disease, including human immunodeficiency infection and COVID-19 (68, 69). Hence, an early alarm and intervention based on carotid ultrasound results might prevent irreversible outcomes of carotid plaques or established CVD.

## Cardiovascular Risk Factors Related to Carotid Atherosclerosis

### Inflammation

Inflammation plays an important role in carotid atherosclerosis. The CIMT and presence of carotid plaques are positively correlated with systematic inflammatory diseases and chronic inflammation (70, 71). Patients with chronic inflammatory disease are at increased risk of cardiovascular events. This is the case for rheumatoid arthritis, the prototype of a chronic inflammatory disease, which is associated with accelerated atherosclerosis (72). Interestingly, several studies have revealed that both CIMT (73) and the presence of carotid plaques (74) are strong predictors of future cardiovascular events in patients with rheumatoid arthritis. Furthermore, carotid ultrasound, as well as other surrogate markers, better identifies rheumatoid arthritis patients with a very high risk of cardiovascular disease than well-defined risk chart algorithms, such as the Systematic Coronary Risk Assessment (SCORE) (75, 76). Moreover, inflammatory intermediate monocytes are reported to correlate strongly with CIMT (77). A proteomic analysis also revealed that CIMT mainly correlates with chemotaxis-related proteins rather than other inflammatory proteins (78). According to the Canakinumab Anti-inflammatory Thrombosis Outcome study (CANTOS), the administration of anti-inflammatory therapy reveals a close association between inflammation and cardiovascular disease (79). An increased inflammatory burden should be carefully considered when carotid ultrasound results are interpreted.

#### Traditional Inflammatory Markers

High-sensitivity C-reactive protein (hsCRP) and serum amyloid A (SAA) are two of the classic acute-phase proteins that have been proven to be independent cardiovascular risk predictors (26, 35). A large asymptomatic cohort revealed a positive association of hsCRP and SAA with the risk for carotid atherosclerotic progression (80). Interestingly, high hsCRP levels predict CVD mortality only in patients with severe atherosclerosis but not in patients with atherosclerosis (44). In addition to hsCRP, other traditional inflammatory markers including fibrinogen and leukocyte counts were demonstrated to be independently associated with the progression of CIMT (81).

#### Cytokines, Chemokines, and Other Novel Inflammatory Factors

A recent systematic review provided a summary of high-risk carotid plaque-related inflammatory cytokines (interleukin-6, interleukin-1β, tumor necrosis factor-α, etc.), chemokines (monocyte chemotactic protein-1, MCP-1), endothelial and cell adhesion factors (intracellular adhesion molecule-1, vascular cell adhesion molecules-1, and selectins), proteolysis factors (matrix metalloproteinases), metabolic biomarkers (lipids, adipokines, homocysteine, etc.), angiogenic markers (vascular endothelial growth factor), and thrombotic biomarkers (plasminogen activator inhibitor-1) (82). Among these serum biomarkers, interleukin 6 was also demonstrated to further increase the predictive capacity, accompanied by the presence of carotid plaques, for obstructive coronary artery disease (83). Other serum inflammatory markers play an important role in both carotid and coronary atherosclerosis. Neopterin, an activation biomarker for monocytes/macrophages, is positively associated with both complex carotid plaques and coronary artery disease (84, 85). High lipoprotein-associated phospholipase A2 (Lp-PLA2), correlated with a high risk of coronary artery disease, is significantly associated with the symptomatic status of carotid plaques (86, 87). Fatty acid binding protein 4, an important inflammatory protein also participating in macrophage cholesterol trafficking, is positively correlated with carotid plaques and stroke symptoms and clearly predicts the risk of cardiovascular mortality (88, 89). Local inflammation of carotid atherosclerosis can be assessed by 2-deoxy-2-[^18^F]fluoro-D-glucose positron emission tomography/computed tomography (^18^F-FDG PET/CT) and the expression inflammatory markers at carotid plaque lesions (86). A high level of galectin 3, a novel vascular inflammatory marker, is a strong predictor of heart failure and poor cardiovascular outcome (90). However, a low intraplaque concentration of galectin-3 is associated with symptomatic and unstable carotid plaques, which can be reversed by short-term statin treatment (91). Serum complement complex C5b-C9 was also an independent risk factor for unstable carotid plaques in patients with acute ischemic stroke (92), whereas the relationship between complement C3 and carotid plaques was controversial in patients with systematic lupus erythematosus (93, 94). Some activated T and B cells, including CD33^+^HLA-DR^+^ T cells, CD19^+^CD86^+^ B cells, CD20^+^CD69^+^ T cells, and CD16^+^ monocytes, were also found to be associated with CIMT, carotid plaques, and the severity of stenosis (95).

Furthermore, moderate doses of statins have been shown to decrease MCP-1 levels followed by CIMT regression, indicating that anti-inflammatory drugs reverse carotid atherosclerosis (96). Therefore, inflammation should be simultaneously assessed with carotid ultrasound for comprehensive evaluations of cardiovascular risk.

### Risk Factors for CVD

#### High-Density Lipoprotein

A low high-density lipoprotein cholesterol (HDL-C) level is well-acknowledged to be associated with high cardiovascular risk, and is also associated with elevated CIMT and carotid plaque burden (97). An elevation in HDL-C levels was shown to be correlated with a reduction in carotid plaque growth in patients with preexisting carotid plaques (98). With the advancement of HDL quality studies, other HDL-related metrics were also found to be associated with carotid atherosclerosis. El Khoudary et al. reported that higher large HDL particles *via* ion-mobility were associated with higher CIMT close to menopause but with lower CIMT in the postmenopausal period (99). Moreover, HDL2-C was positively associated with carotid plaque thickness, while HDL3-C was inversely associated with carotid plaque area (100). The relationship between protein components in HDL and carotid atherosclerosis was also investigated. Aroner et al. found that HDL containing apoC-III was positively associated with carotid plaque score, while HDL lacking apoC-III was negatively associated with carotid plaque score and CIMT, which supported the role of apoC-III in HDL in carotid atherosclerosis (101). Surprisingly, Shea et al. reported that HDL-mediated cholesterol efflux capacity was positively associated with carotid plaque progression, but negatively associated with incident hard CVD based on cross-sectional analysis (102). The correlation between HDL function and carotid atherosclerosis requires further investigation. Non-HDL-C, calculated as total cholesterol minus HDL-C, and the non-HDL-C/HDL-C ratio were also cardiovascular risk factors positively associated with carotid atherosclerosis (103, 104). In total, HDL-related metrics were important variables for cardiovascular risk prediction and the evaluation of carotid atherosclerosis.

#### Low-Density Lipoprotein

Low-density lipoprotein cholesterol (LDL-C) is a strong predictor for both cerebrovascular events and cardiovascular events, and the recommended target of LDL-C becomes lower in order to reduce residual risk (105). A greater possibility of a higher carotid plaque score was demonstrated to be related to an increase in LDL-C within 1 year of the final menstrual period, which might be associated with an elevation of cardiovascular risk for postmenopausal women (106). Furthermore, an increased circulating oxidized LDL-C was significantly associated with a higher risk of 10-year progression of subclinical carotid plaques (107). However, a targeted LDL-C level of <70 mg/dL did not reduce the incidence of newly diagnosed carotid plaque compared to a higher LDL-C target in patients with ischemic stroke (108). Therefore, early target control of LDL-C before the incidence of atherosclerotic events is more beneficial in cardiovascular prevention.

#### Diabetes

Diabetes is one of the strongest risk factors for both carotid atherosclerosis and CVD, and the detection of carotid plaque is recommended in diabetic high-risk patients (39, 109). The presence of echogenic carotid plaques, compared to that of echolucent and heterogeneous plaques, was demonstrated to be a stronger predictor for incident major adverse cardiovascular events in patients with type 2 diabetes (110). Patients with type 1 diabetes also had a higher proportion of echogenic and calcified plaques than subjects without type 1 diabetes (111). Furthermore, the frequency of carotid plaques was increased in patients with latent autoimmune diabetes in adults (LADA) compared to type 1 and type 2 diabetes, which was also increased with increasing diabetes duration in LADA (112). For diabetic complications, obesity, renal function decline, and diabetic retinopathy were investigated to be positively associated with the presence of carotid plaques (113–115). Therefore, carotid ultrasonography is necessary for the evaluation of vascular complications as well as the risk of cardiovascular events.

#### Hypertension

Hypertension is an important traditional risk factor, and antihypertensive targeted therapy is protective against cardiovascular events (116). Both high systolic and diastolic blood pressure at age 40 were demonstrated to be associated with carotid plaque burden late midlife (117). Additionally, carotid plaque score and CIMT were demonstrated to be potent predictors for stroke, and the former performed more accurately (118). H-type hypertension, characterized by hypertension and hyperhomocysteinemia with high cardio-cerebrovascular risk, was reported to be positively associated with higher presence of carotid plaques than isolated systolic hypertension and simple hypercysteinemia (119). Recently, Ben et al. also found that blood homocysteine levels in hypertensive patients with hyperhomocysteinemia were positively associated with carotid plaque thickness, stenosis degree, and contrast-enhanced ultrasound quantification, but negatively associated with shear wave velocity (120). Hence, carotid ultrasonography is a useful tool for atherosclerotic evaluation of hypertensive patients.

#### Unhealthy Lifestyle

An unhealthy lifestyle associated with increased cardiovascular risk can also promote carotid atherosclerosis. Smoking is one of the major atherosclerotic factors, and both current and former smokers were at higher risk of echodense carotid plaques than never smokers (121). Sedentary behavior is another common unhealthy lifestyle. A moderate level of physical activity with a sedentary time ≤ 3 hours/day was associated with lower odds of the presence of carotid plaques, but no reduction in carotid plaque presence by physical activity combined with a sedentary time >3 hours/day (122). Moreover, a Western dietary pattern, including higher red meat, sugar intake, and deep-fried products, was positively associated with higher CIMT in the common carotid artery, which might contribute to future cardiovascular risk (123). Poor sleep quality and short sleep time, a universal phenomenon of menopause, were found to be associated with higher CIMT and odds of carotid plaques (124). Middle-aged male shift workers also had higher CIMT and carotid plaque presence than fixed daytime workers (125). Additionally, sleep apnea, defined as an apnea-hypopnea index of 15 events per hour, was associated with an increased presence of carotid plaque in subjects aged <68 years but not in older adults. Greater hypoxemia was also associated with increasing carotid intima-media thickness in younger subjects but not in older adults (126). Patients with chronic obstructive pulmonary disease had higher CIMT (127), and lower pulmonary function was associated with an increased risk of carotid atherosclerosis compared to higher pulmonary function (128).

#### Intervention for Traditional Risk Factors

Traditional cardiovascular risk factors, including age, sex, blood pressure, smoking history, lipid levels, and diabetes, are independent determinants of the presence of carotid atherosclerosis (129–131). Previous evidence has shown that traditional risk factors do not largely contribute to the variance in CIMT and carotid plaque burden (132, 133). CVD risk factors have also been reported to partly account for of the total carotid plaque area and three-dimensional carotid plaque volume (129, 134). However, an improvement in CVD risk factors may not reduce carotid plaque progression to a greater extent than an early increase in the CIMT. As mentioned above, an early treat-to-target approach for CVD risk factors has been shown to slow down CIMT progression and reduce the risk of incident cardiovascular events in a high CIMT population without prior CVD (66). However, no benefits of exercise training in terms of CIMT reduction were observed in patients with established CVD (67). Moreover, in the Study of Women's Health Across the Nation, a healthier diet score was associated with a smaller CCA-IMT and CCA adventitial diameter, but it was not significantly correlated with the presence of carotid plaques (135). Our previous network meta-analysis also demonstrated that high-intensity statins and the combination therapy of statins and ezetimibe were associated with larger CIMT reduction compared to moderate/low-intensity statins and no statins, but the evidence for the association between statins and carotid plaque changes remained insufficient (136). Another population-based observational study did not observe a correlation between omega-3 fatty acid consumption and the presence of carotid plaques (137).

Hence, CVD risk factors interact with carotid atherosclerosis, and treatments targeting CVD factors may possibly reverse carotid atherosclerosis in the early phase, but multidisciplinary efforts are needed for the early prevention of carotid atherosclerosis progression.

## Evaluation of Carotid Ultrasonography Combined With Cardiovascular Risk Factors

**Figure 3:**
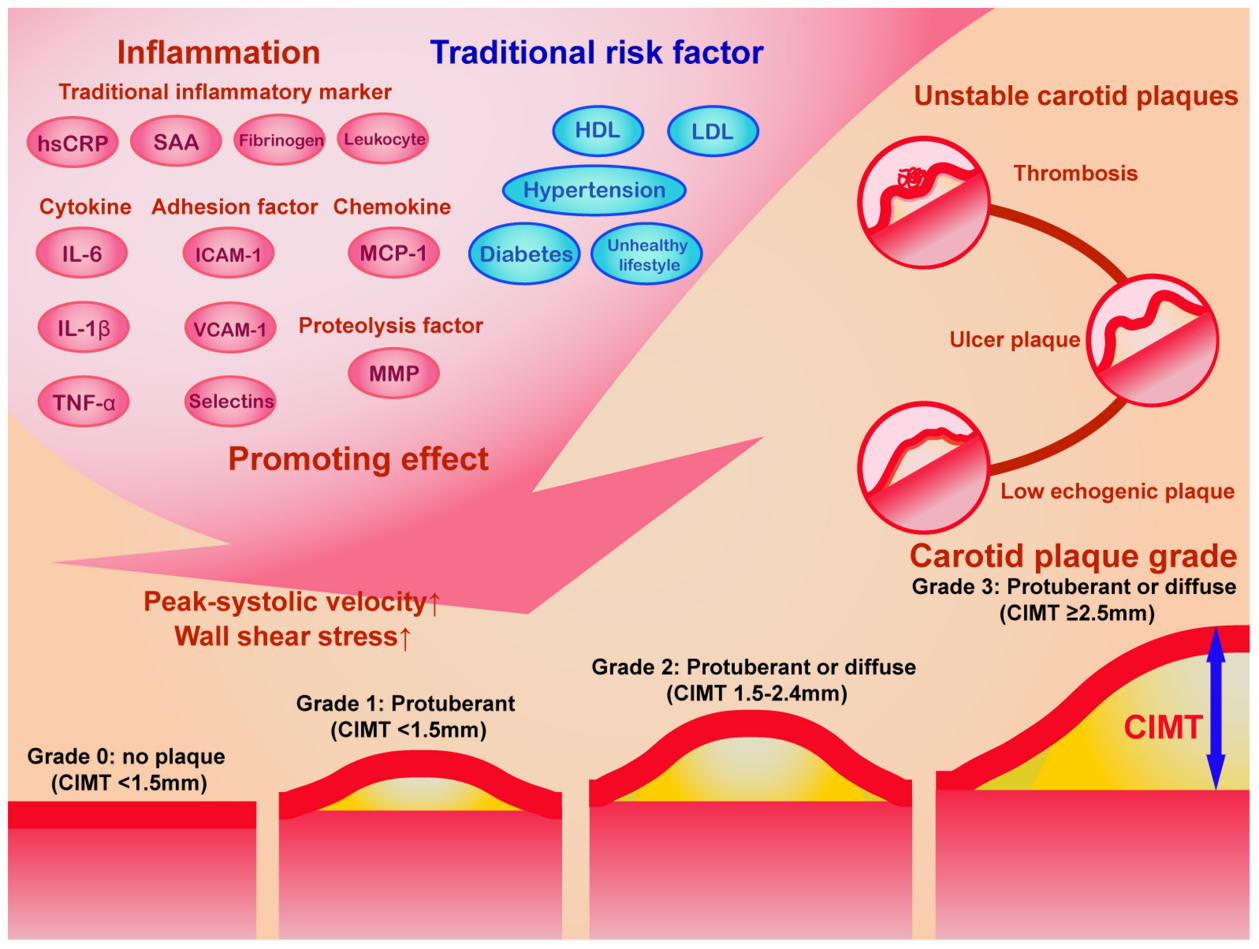
The schematic diagram of the impact of inflammation and traditional risk factor on carotid plaque progression and the grading system of carotid plaques. Traditional inflammatory markers, cytokines, chemokines, adhesion factors, proteolysis factors, and other novel inflammatory factors, in combination with traditional risk factors promotes elevated carotid plaque grade and unstable carotid plaques. According to Recommendations for the Assessment of Carotid Arterial Plaque by Ultrasound from the American Society of Echocardiography in 2020 (41), the carotid plaque grade was classified into 4 levels: grade 0 (no plaques with CIMT <1.5 mm), grade 1 (protuberant CIMT < 1.5 mm), grade 2 (protuberant or diffuse CIMT between 1.5 and 2.4 mm), and grade 3 (protuberant or diffuse CIMT ≥2.5 mm). The ultrasound characteristics of unstable carotid plaques includes low echogenic plaques, ulcer plaques, and thrombosis. CIMT, carotid intima-media thickness; HDL, high-density lipoprotein; hsCRP, hypersensitive C reactive protein; ICAM-1, intercellular cell adhesion molecule-1; IL-6, interleukin-6; IL-1β, interleukin-1 beta; LDL, low-density lipoprotein; MCP, monocyte chemotactic protein-1; MMP, matrix metallopeptidase; TNF-α, tumor necrosis factor-alpha; VCAM-1, vascular cell adhesion molecule-1.

**Figure 4:**
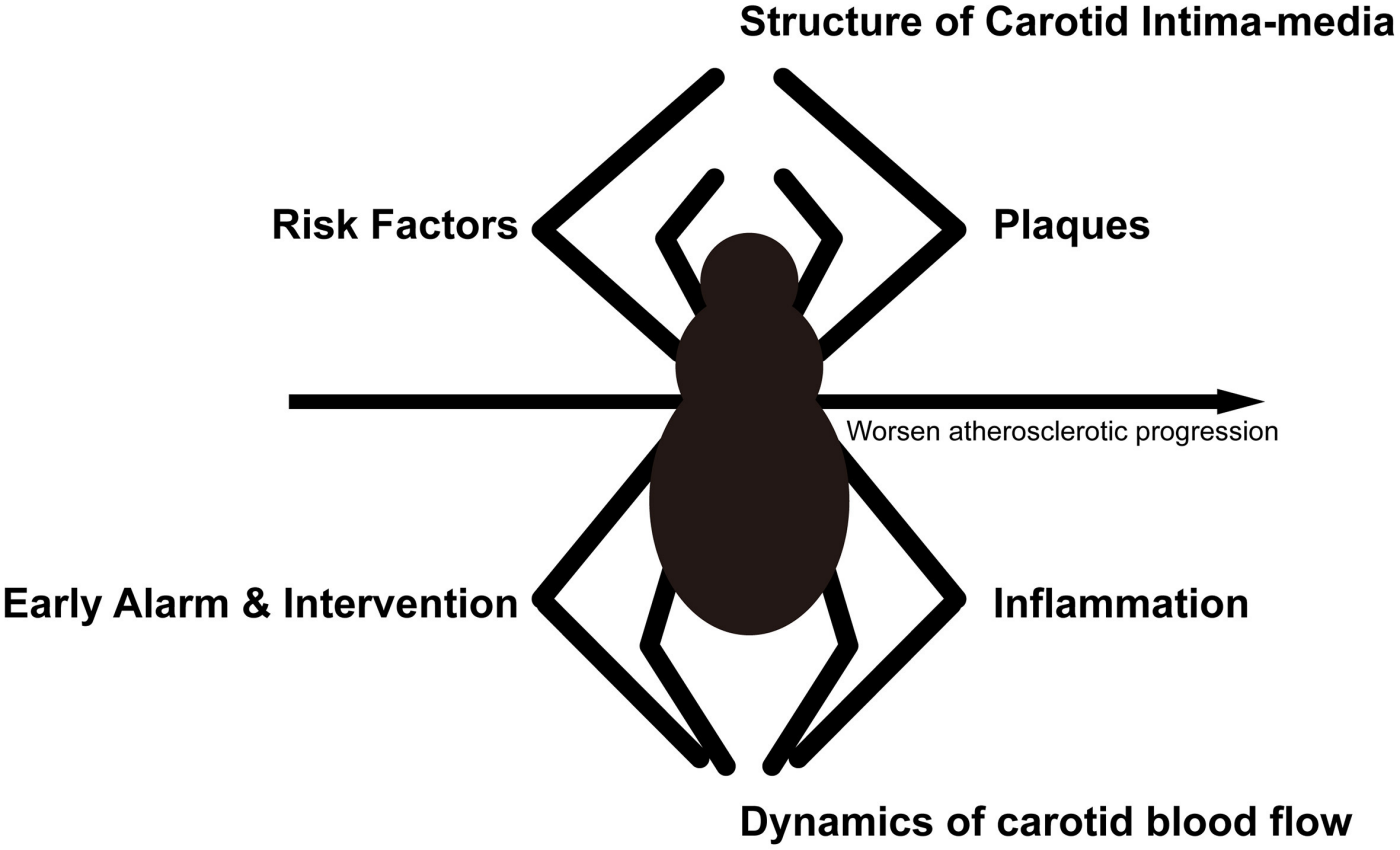
Schematic diagram of the “SPIDER” proposal. The progression of atherosclerosis is similar to a spider spinning a web, and a full consideration of these 6 aspects may help to prevent the progression of atherosclerosis.

The prevalence of carotid atherosclerosis increases with age (39, 138); thus middle-aged and elderly individuals are at higher risk of carotid atherosclerosis and CVD than young adults and may benefit from carotid ultrasound detection. Inflammatory factors and traditional risk factors have integral effect on the hemodynamics and vessel dysfunction of carotid artery, which causes carotid plaque progression and instability (Figure 3). Additionally, high-risk plaque is not clearly defined, and lacks evidences for intervention, although it is largely correlated with cerebrovascular and cardiovascular disease. Several serum atherosclerotic biomarkers combined with carotid ultrasonography may assist clinicians in identifying high cardiovascular risk patients who need intervention (82). When evaluating the risk of CVD using carotid ultrasonography, the major problem is the lack of normative values of carotid ultrasound parameters and the weak combination of carotid ultrasound results with cardiovascular risk factors in clinical practice. Based on the comprehensive literature review and within the context of international guidelines (1, 33, 34, 139), we propose that atherosclerosis, like a web, is promoted and alarmed by the “SPIDER” (Figure 4), the name of which originated from the first letter of the 6 abovementioned aspects. A comprehensive assessment of combining carotid ultrasonography and other atherosclerotic factors may halt the progression of atherosclerosis for cardiovascular prevention.

## Conclusion

Carotid ultrasound results should be combined with other important atherosclerotic factors, and a comprehensive cardiovascular assessment can better predict CVD risk and guide primary preventative measures.

## Author Contributions

HL and XX contributed to literature review and manuscript drafting. BL contributed to providing image. YZ contributed to the conception of the work and assessed the quality of evidence and suggestions. All authors contributed to the article and approved the submitted version.

## Conflict of Interest

The authors declare that the research was conducted in the absence of any commercial or financial relationships that could be construed as a potential conflict of interest.

## Publisher's Note

All claims expressed in this article are solely those of the authors and do not necessarily represent those of their affiliated organizations, or those of the publisher, the editors and the reviewers. Any product that may be evaluated in this article, or claim that may be made by its manufacturer, is not guaranteed or endorsed by the publisher.
